# Ⅰ期非小细胞肺癌术后辅助化疗的研究进展

**DOI:** 10.3779/j.issn.1009-3419.2015.06.08

**Published:** 2015-06-20

**Authors:** 博 刘, 凤霞 丁, 双强 杨

**Affiliations:** 400016 重庆，重庆医科大学附属第一医院胸心外科 Department of Cardiothoracic Surgery, The First Affiliated Hospital of Chongqing Medical University, Chongqing 400016, China

**Keywords:** 肺肿瘤, Ⅰ期, 术后辅助化疗, Lung neoplasms, Stage Ⅰ, Postoperative adjuvant chemotherapy

## Abstract

肺癌发病率及死亡率居全球癌症首位，按组织病理学可分为非小细胞肺癌（non-small cell lung cancer, NSCLC）和小细胞肺癌。仅有不到20%的NSCLC患者在初次诊断时有手术机会，而术后5年生存率大约只有30%-60%，单纯手术治疗的效果难以令人满意。很多临床随机试验结果显示术后辅助化疗可使Ⅱ期-Ⅲa期NSCLC患者生存获益，而Ⅰ期NSCLC患者是否需要术后辅助化疗存在争议。本文就Ⅰ期NSCLC术后化疗人群选择的影响因素、化疗方案的选择以及生物学标志物等方面的研究进展做一综述。

## Abstract (en)

The morbidity and mortality of lung cancer rank the first place among all the malignant tumor. According to the histopathological characteristics, lung cancer is divided into non-small cell lung cancer (NSCLC) and small cell lung cancer. Only 20% patients diagnosed with NSCLC have the chance for surgery while their 5-yr overall survival is about 30%-60%. The therapeutic outcome of surgery alone is not satisfying. Adjuvant chemotherapy after surgical resection instage Ⅱ-Ⅲa lung cancer showed efficacy in many randomized clinical trials, but its role instage Ⅰ disease remains controversial. The choice of appropriate chemotherapy candidates, the selection of chemotherapy regimens and the research progress on biomarker are mainly discussed in this review.

最新数据^[1]^显示，肺癌发病率和死亡率均居恶性肿瘤首位，其可分为非小细胞肺癌（non-small cell lung cancer, NSCLC）和小细胞肺癌，其中NSCLC占80%-85%。外科手术是NSCLC最有效的治疗手段。但令人遗憾的是，NSCLC患者术后5年生存率大约只有30%-60%，肿瘤复发和转移是导致术后生存率偏低的主要原因，虽然Ⅰ期NSCLC患者相对来说预后更好，但是Ⅰ期患者仅行手术后局部复发率约6%-11%，远处转移率约23%-30%^[2]^。术后辅助化疗可使Ⅱ期-Ⅲa期NSCLC患者5年生存率提高4%-15%^[2-4]^，但对于Ⅰ期NSCLC患者是否需要术后辅助化疗至今尚无统一定论。

## 1 影响Ⅰ期NSCLC术后化疗人群选择的因素

### 1.1 影响Ⅰ期NSCLC术后化疗人群选择的病理因素

#### 1.1.1 肿瘤-淋巴结-转移（tumor-node-metastasis, TNM）分期

目前的TNM分期（2009版）将T1细分为T1a（≤2 cm）和T1b（3 cm≥T > 2 cm），T2细分为T2a（5 cm≥T > 3 cm）和T2b（7 cm≥T > 5 cm），T2bN0M0由Ⅰb期升为Ⅱa期，而T > 7 cm的T2变为T3，这对术后化疗人群的选择产生了新的影响。Li^[5]^按照目前的TNM分期对1998年-2003年间325例手术完整切除的Ⅰ期NSCLC患者预后重新进行评价，肿瘤直径≤2 cm和2 cm < 肿瘤直径≤3 cm患者5年生存率分别为75.49%和74.58%，3 cm < 肿瘤≤5 cm或5 cm < 肿瘤≤7 cm患者5年生存率分别为60.87%和55.63%，而肿瘤直径 > 7 cm的患者5年生存率仅为46.15%，肿瘤大小是Ⅰ期NSCLC的独立预后因素。目前美国国立综合癌症网络（National Comprehensive Cancer Network, NCCN）指南推荐对肿瘤直径≥4 cm的Ⅰb期NSCLC患者术后辅助化疗，依据是CALGB 9633试验^[6]^亚组分析结果；Malhotra^[7]^对2, 999例Ⅰ期NSCLC研究后同样认为，直径≥4 cm的NSCLC患者术后辅助化疗能够生存获益。但近期发布的Lungscape报告^[8]^，对15个中心的2, 449例已行手术切除治疗的Ⅰ期-Ⅲ期NSCLC患者研究后发现，术后辅助化疗未能改善肿瘤 > 4 cm患者的预后，因此，肿瘤直径4 cm是否是合适的化疗人群选择分界点还有待进一步研究。

#### 1.1.2 肿瘤所在部位

Ou等^[9]^分析了19, 702例Ⅰ期肺癌患者，发现Ⅰ期NSCLC非上叶患者（右中叶、右下叶、左下叶）较上叶患者死亡风险增加，虽然上叶NSCLC发生纵隔淋巴结转移的概率相对较高，但是其纵隔淋巴结转移往往局限于上纵隔区域淋巴结，而中下肺叶NSCLC则易出现上、下纵隔跳跃式淋巴结转移。Kudo等^[10]^认为左肺叶肺癌的预后较其他肺叶差，左肺肿瘤是影响预后的独立危险因素，这可能是由于左肺癌可以同时通过同侧和对侧纵隔淋巴结转移，而右肺癌主要通过同侧纵隔淋巴结进行转移。因此，肿瘤所处肺叶部位可能会影响患者的预后，从而影响化疗人群的选择。

#### 1.1.3 组织病理学类型和分化程度

Mediratta等^[11]^认为Ⅰ期NSCLC患者预后与组织病理学类型有关，腺癌患者预后较差。相比其他组织病理学类型而言，鳞癌发生转移相对较晚，而腺癌的肿瘤细胞更容易通过血液及淋巴结向其余器官进行扩散^[12]^。不同组织病理学类型的NSCLC发生远处转移的概率不同，可能影响患者术后辅助化疗的预后。而组织分化程度也与预后密切相关，即使是早期NSCLC，分化程度差的患者也需行术后辅助化疗。Kuo等^[13]^对影响Ⅰ期NSCLC无进展生存期的病理因素进行了探索，发现组织分化程度低是Ⅰ期NSCLC患者术后复发的危险因素，Maeda等^[14]^也认为分化程度差预示着Ⅰ期肺癌恶性程度高，容易早期出现血管及淋巴转移，辅助化疗可以使术后复发风险降低。

#### 1.1.4 淋巴血管浸润及脏层胸膜受累情况

一项研究^[15]^比较了Ⅰ期患者有或无淋巴血管浸润的生存情况，结果显示有淋巴血管浸润的患者术后复发风险升高，淋巴血管浸润可作为NSCLC转移的早期表现，术后辅助化疗可以减少这种早期微转移灶。而脏层胸膜受侵则会增加T分期，即使直径≤3 cm的肿瘤也会从Ia期变为Ⅰb期，术后局部复发风险明显增加，因此，这部分患者推荐给予术后辅助化疗。Yanagawa等^[16]^对433例Ⅰ期肺癌术后患者进行回顾性分析，发现脏层胸膜及淋巴血管侵犯影响患者预后，两者与肿瘤复发的部位和复发的概率有一定关系，可以作为判断术后是否需要辅助化疗的一个候选的标记。另外，脏层胸膜受侵只可以用来评估实性小结节肺癌患者的术后生存情况，在肺部磨玻璃影（ground-glass opacity, GGO）患者中，如果脏层胸膜受侵不能因此改变TNM分期而行术后辅助化疗^[17]^。

### 1.2 影响Ⅰ期NSCLC术后化疗人群选择的临床因素

#### 1.2.1 体能状态（performance status, PS）评分

临床上常用PS评分来评估患者术后的身体情况，判断其对辅助化疗的承受能力，已经证实0分-1分的患者可从辅助化疗中获益^[3]^，PS刚好2分的患者使用单药化疗或联合化疗有一定的风险，2分以上患者不建议使用细胞毒性药物，以最佳支持治疗为主^[18]^。

#### 1.2.2 合并症

肺癌患者多合并有其他系统疾病，而charlson评分可以用来评估合并症的严重程度，有学者认为charlson评分越高，表示患者合并症多且严重，生存情况越差；因此有很大一部分有合并症的患者术后延长开始治疗的时间，减量化疗，甚至未接受化疗。但根据最新的Ontario癌症登记处的研究报告，没有证据证明患者合并症越多，则需要延长术后辅助化疗开始的时间，也没有发现术后辅助化疗的开始时间与生存期有必然的联系。因此，合并症不严重或charlson评分3分及以下的患者术后应按时化疗^[19]^。

#### 1.2.3 年龄

在临床上，很多老年患者因为担心承受不了化疗的毒副反应及化疗效果得不到保障而放弃术后辅助化疗。但有学者认为老年NSCLC患者同样可以从术后辅助化疗中获益，Wisnivesky等^[20]^随访了3, 324例65岁以上Ⅱ期-Ⅲa期NSCLC患者术后生存情况，其中684例患者术后接受含铂方案化疗，结果显示 < 70岁和70岁-79岁之间的患者术后辅助化疗能够生存获益，而80岁以上患者未见生存获益。同样，Cuffe等^[21]^评估了2, 763例70岁以上的NSCLC患者术后接受顺铂或卡铂为基础化疗的效果，有70岁-74岁、75岁-79岁和≥80岁这三个年龄组，结果提示化疗后老年NSCLC患者生存期有明显改善，80岁以上年龄组患者除外。因此，老年肺癌患者术后不应放弃辅助化疗，80岁以下的老年NSCLC患者术后同样需要辅助化疗^[22]^。

#### 1.2.4 性别

针对预后的临床研究比较了不同性别人群的生存差异。有研究^[23]^显示，女性NSCLC患者预后较好，这可能与女性患者术后较少出现淋巴结转移，同时雌激素水平、基因以及情绪因素可能在其中起了重要作用，但性别是否为独立预后因素还有待于进一步研究^[24]^。

#### 1.2.5 血清癌胚抗原（carcino-embryonic antigen, CEA）水平

CEA水平有助于揭示影像学及病理检查所不能发现的微转移灶及残存的肿瘤细胞，给NSCLC患者术后是否需要化疗提供参考依据。Wang等^[25]^发现术后CEA水平可以用来判断Ⅰ期NSCLC患者预后，术后CEA水平持续升高者其预后较差，更需要考虑辅助化疗。Yamazaki等^[26]^的研究也显示血清中CEA高与肿瘤的侵袭性和预后差有关，术后CEA水平可以作为Ⅰ期NSCLC患者术后辅助化疗人群选择的参考指标，有人将正常高值定为7.0 ng/mL^[27]^。

#### 1.2.6 影像学检查

高分辨率计算机断层扫描（computed tomography, CT）可以清楚显示病灶密度改变、边缘情况及邻近血管、胸膜的情况；肿瘤直径偏大，实性成分百分比例高，毛刺征和胸膜凹陷可以预测早期肺癌术后容易复发。Ⅰ期NSCLC如果有空洞形成，预示着肿瘤恶性程度较高，生长速度超过肿瘤血供所能承受的范围而出现液化坏死^[23]^。CT肿瘤形态学有上述改变的NSCLC患者需要选择术后辅助化疗。正电子发射断层显像/计算机断层扫描（positron emission tomography/computed tomography, PET/CT）在临床上的应用也越来越多，Kishimoto等^[28]^发现术前PET/CT的SUVmax在预测术后复发风险方面优于薄层CT的C/T比（肿瘤实性成分的最大直径与肿瘤的最大直径之比），SUVmax≥2.5的患者无病生存期降低，通过SUVmax值预测小体积肺癌的侵袭性和预后很有必要。Sasada等^[29]^认为可根据SUVmax值来选择Ⅰ期NSCLC患者化疗的优势人群，SUVmax≥2.6的患者术后辅助化疗可以生存获益。另外，影像学检查还可以发现术后新出现的纵隔或远处转移灶，可以用来判断化疗药物的疗效并指导制定合适的化疗方案。

### 1.3 手术因素对Ⅰ期NSCLC术后化疗人群选择的影响

#### 1.3.1 手术方式

Varlotto等^[30]^随访了411例接受手术治疗的Ⅰ期NSCLC患者，中位随访时间为34个月，分为亚肺叶切除组和肺叶切除组，比较两组患者的预后，亚肺叶切除组患者局部复发的风险增加，在中等分化或肿瘤 > 2 cm的患者中表现得更为明显，因此，辅助化疗前接受不同的手术方式也会对患者的预后产生影响；结合Tsutani等^[31]^的临床研究提到术后辅助化疗对 > 2 cm的Ⅰ期NSCLC患者有效，在临床上，对肿瘤 > 2 cm的患者施行亚肺叶切除术后，可考虑给予辅助化疗，以降低肿瘤复发风险。

#### 1.3.2 手术切缘

El-Sherif等^[32]^随访了81例接受亚肺叶切除术的患者，41例切缘距离肿瘤 < 1 cm的患者，最终有6例患者进展为局部复发；而40例肿瘤切缘≥1 cm的患者中，仅3例患者局部复发，证实了肿瘤病灶完整切除的重要性。另外，中国版NCCN指南也将切缘 < 1 cm患者定为术后复发高危人群，建议选择辅助化疗。

#### 1.3.3 术中淋巴结处理方式

肺癌手术中淋巴结的处理方式很多，纵隔淋巴结系统切除术（mediastinal lymph node dissection, MLND）与纵隔淋巴结采样（systematic sampling, SS）是当前使用最多的两种处理方式，过去的观点认为系统淋巴结清扫可以改善NSCLC患者术后生存情况，但ACOSOG Z0030试验^[33]^和最新的一项*meta*分析^[34]^结果显示纵隔淋巴结切除术组和纵隔淋巴结采样组患者的总生存率、局部复发率以及远处转移率相似；但值得注意的是，即使亚厘米的实性肺癌仍可以通过淋巴结进行转移^[35]^，系统淋巴结清扫术可以提高分期的精度并达到根治的目的，如果术中仅行纵隔淋巴结采样，建议术后辅助化疗。

## 2 化疗方案的选择

在临床中多选用患者依从性较好及毒性反应较小的化疗方案。美国国立综合癌症网络（National Comprehensive Cancer Network, NCCN）推荐的化疗方案有：①顺铂联合培美曲塞，主要适用于非鳞癌的患者；②顺铂联合吉西他滨，主要适用于鳞癌患者；③顺铂联合其他第三代化疗药物如紫杉醇、多西他赛等；④对于老年晚期NSCLC患者等合适的情况，使用培美曲塞等单药化疗也是可以的。此外，还有部分学者使用顺铂/长春瑞滨化疗方案，但是由于长春瑞滨的骨髓抑制作用较明显，因此在临床上该方案已少用。辅助化疗一般使用4个-6个周期，根据患者身体状况及化疗药物疗效等情况继续调整。日本的一项研究^[36]^进行了紫杉醇/卡铂和吉西他滨/卡铂对完整切除术后NSCLC辅助化疗疗效比较，主要终点指标是依从性，次要终点指标是安全性和毒性，试验结果证实两种化疗方案中患者依从性好，化疗方案切实可行，毒性在可接受的范围内。另外，Kreuter负责的TREAT^[37]^临床试验，比较了长春瑞滨/顺铂和培美曲塞/顺铂方案化疗疗效，主要终点指标为临床可行性，结果显示培美曲赛联合顺铂方案安全可行，毒性小于长春瑞滨/顺铂方案，化疗完成情况优于长春瑞滨/顺铂方案。2014年欧洲临床肿瘤协会年会（European Society for Medical Oncology, ESMO）上展示了一项比较培美曲塞/顺铂（AP）或多西他赛/顺铂（DP）治疗非鳞状NSCLC患者疗效的Ⅲ期临床试验结果（LBA41_PR），共有149例患者进入研究，主要比较两组患者的无进展生存期（progression-free survival, PFS）有无差别，同时观察两组的缓解率、化疗药物安全性等，研究显示两组无进展生存期及缓解率无明显差异，但是DP方案化疗副反应相对更大。

优福定（Uracil-Tegafur, UFT）是替加氟（FT-207）与尿嘧啶（Uracil）按分子量计算以1:4混合制成的口服制剂，FT-207通过羟化在体内转化为5-氟尿嘧啶，尿嘧啶抑制二氢吡啶脱氢酶而调节5-氟尿嘧啶的代谢，使其有效浓度在体内维持较长时间，从而增强抗肿瘤的生物活性^[38]^。日本的一项*meta*分析^[39]^共纳入6项研究，包含了2, 003例患者，其中大多数为Ⅰ期（96%）和腺癌患者（85%），合并分析显示术后UFT辅助化疗能使Ⅰ期患者生存获益（HR=0.74, 95%CI: 0.61-0.88; *P*=0.001），特别是肺腺癌患者；结论是日本Ⅰ期NSCLC患者术后口服1年-2年UFT是有效的；在日本，UFT被推荐作为肿瘤 > 2 cm的Ⅰ期NSCLC患者术后标准治疗方案，但是UFT目前在欧美认可度不高，在我国利用UFT化疗也鲜有学者报道。因此，同样作为亚洲人群，中国肺癌患者是否也对UFT敏感，尤其是腺癌患者的化疗方案是否需要加用该药，仍需要进一步研究。

## 3 生物学标志物

切除修复交叉互补基因1（excision repair cross complementary gene 1, *ERCC1*）位于人体的19号染色体，参与DNA链的切割和损伤识别；核苷酸还原酶1（ribenucleotide reductase M1, RRM1）的表达可减缓肿瘤细胞DNA的复制，两者的表达具有明显的相关性。Bepler等^[40]^对IALT试验中784例患者术后肿瘤组织ERCC1和RRM1进行检测，发现含铂方案化疗组中ERCC1低表达患者OS及DFS均优于高表达患者，低RRM1表达患者同样有获益趋势，如果检测出两者高表达，不建议使用铂类为基础的化疗。

乳腺癌易感基因1（breast cancer susceptibility gene 1, *BRCA1*）位于人类17号染色体，在基因的表达中有很高的外显率，同时在化疗介导的细胞凋亡中具有重要作用，BRCA1所在DNA转录而来的信使RNA携带相关遗传信息在人体中进行表达。大量临床研究^[41, 42]^结果提示，如果该基因外显率越高，则出现使用铂类药物化疗无效的可能性越大；*BRCA1*基因在肺癌患者施行铂类辅助化疗的敏感性预测中可能起着一定作用。另外，抗微管类药物可与β微管蛋白Ⅲ（class Ⅲ beta-tubulin, TUBB3）结合，通过阻止微管蛋白的聚合或解聚来抑制细胞有丝分裂的进行，从而诱导细胞凋亡。Hirai等^[43]^发现TUBB3高表达患者对长春瑞滨化疗的敏感性更好，一项*meta*分析^[44]^纳入10项研究共552例患者，合并分析数据显示紫杉醇/长春瑞滨化疗组中TUBB3阴性或少表达的患者客观缓解率更高，中位生存时间更长。

胸苷酸合成酶（thymidylate synthase, TS）可以调控腺苷酸的组合速度，在核酸的合成中起着重要作用。2014年ESMO上报告了一项Ⅱ期试验结果（LBA42_PR），315例非鳞状NSCLC患者根据TS阳性（超过10%的肿瘤细胞表达TS）或TS阴性，分成顺铂/培美曲塞组和顺铂/吉西他滨组，研究结果显示，对于TS阴性的患者，两组患者的治疗有效率为47.0%和21.1%；而对于TS阳性的患者，两组患者的治疗好转率为40.3%和39.2%。如果TS阴性表达，接受两组不同化疗方案患者的中位DFS分别为6.4个月和5.5个月，TS阳性表达，接受两种不同化疗方案患者的中位DFS分别为5.9个月和5.3个月。提示TS的表达水平可用于预测非鳞癌NSCLC化疗效果，TS阴性患者使用培美曲塞/顺铂化疗能够延长术后生存时间。

*P53*是一种抑癌基因，在肿瘤的形成中起着重要的作用。Tsao^[45]^对JBR.10^[46]^肿瘤标本中的P53基因/蛋白畸变的预后及预测价值进行评估，发现其中132例患者P53蛋白过表达呈阳性，单纯手术组的P53阳性患者总生存期相比化疗组P53阴性患者明显缩短，而在辅助化疗组中P53阳性患者相比P53阴性患者能够从辅助化疗中明显获益，P53蛋白过表达是生存期缩短的预后标志物，同时也是肺癌术后辅助化疗生存获益的重要标记。

表皮因子生长受体（epidermal growth factor receptor, EGFR）在调控细胞增殖中起着重要作用。Izar等^[47]^对完整切除术后的Ⅰ期NSCLC患者组织标本中的*EGFR*的突变状态进行检测，包括EGFR突变及EGFR野生型，比较两者的复发率、无病生存率及总生存率，结果显示，*EGFR*突变相比*EGFR*野生型肺癌患者，复发率降低，中位DFS延长，5年OS提高，*EGFR*突变阳性患者预后更佳。因此，*EGFR*基因突变阳性可作为NSCLC患者一个积极的预后指标。Capelletti等^[48]^对CALGB 9633^[6]^试验中化疗组患者组织标本中的KRAS基因进行了检测，研究结果显示即使肿瘤大小超过4 cm，如果存在该基因突变，辅助化疗也不能使患者生存期延长。Tsao^[45]^检测了JBR.10肿瘤标本中*KRAS*基因突变情况，发现如果是野生型*KRAS*表达，术后行辅助化疗能够延长患者的OS，而存在*KRAS*突变的患者术后行辅助化疗OS未见延长。此外，近年来新发现了多个与NSCLC预后及化疗效果相关的基因，如*ALK*、*ROS1*等^[49]^，通过对这些基因的检测，有助于选择合适的化疗优势人群，预测化疗药物的疗效，从而达到个体化治疗目的。

值得注意的是，生物学标志物目前尚存在样本处理不够标准化、检测方法不可靠及抗体的特异性差、mRNA和蛋白检验的异质性等问题。例如，Wislez等^[50]^设计Ⅱ期临床试验（IFCT-0801）中探讨基于ERCC1表达的个体辅助治疗用于早期NSCLC患者的疗效，发现同一样本两次测得的ERCC1外显率却不相同，这与抗体识别基因表达蛋白特异性较差有关，最终该试验被迫停止。备受关注的RADIANT（NCT00373425）试验^[51]^旨在验证厄洛替尼治疗Ⅰb期-Ⅲa期EGFR阳性NSCLC患者的临床效果，973例患者随机分为厄洛替尼组和安慰剂组，研究结果显示，厄洛替尼未能延长NSCLC患者的无进展生存期，厄洛替尼对EGFR突变患者的临床效果需进一步研究。欧洲肿瘤内科学会（European Society for Medical Oncology, ESMO）暂不推荐临床上对生物学标志物进行常规检测来判断预后、指导治疗方案及预测化疗药物的效果^[52]^。但从另一方面看，NSCLC是一种分子生物学上高度特异性的疾病，生物学标记将有助于从基因水平判断患者的预后和筛选合适的化疗人群，指导化疗方案的选择及判断化疗方案的疗效，越来越多的肺癌患者有望从个体化辅助治疗中获益。

## 4 小结

目前NCCN指南只推荐对Ⅰb期NSCLC完整切除术后复发高危人群进行辅助化疗（2A证据），包括分化水平差、血管受侵、楔形切除、肿瘤直径 > 4 cm、脏层胸膜受累及局部淋巴结无法评估（Nx）的患者，而Ia期NSCLC患者不推荐术后辅助化疗。由于缺乏足够的循证医学证据作为支持，可能未考虑到所有的复发高危人群。化疗对象应该在TNM分期基础上结合患者自身临床病理因素进行选择。化疗方案首选顺铂联合培美曲塞、吉西他滨、紫杉醇、多西他赛等第三代化疗药物。术后辅助化疗同样适用于体能状况较好的老年NSCLC患者，但80岁以上患者术后辅助化疗需慎重，生物学标志物用来判断患者的预后和筛选合适的化疗人群、指导化疗方案的选择及判断化疗方案的疗效有待于进一步研究。
